# Entropy and the Direction of Time

**DOI:** 10.3390/e23040388

**Published:** 2021-03-25

**Authors:** Jerzy Gołosz

**Affiliations:** Institute of Philosophy, Jagiellonian University, ul. Grodzka 52, 31-044 Kraków, Poland; jerzy.golosz@uj.edu.pl

**Keywords:** asymmetry of time, asymmetry in time, direction of time, entropy, Boltzmann, Reichenbach, David Z. Albert

## Abstract

The paper tries to demonstrate that the process of the increase of entropy does not explain the asymmetry of time itself because it is unable to account for its fundamental asymmetries, that is, the asymmetry of traces (we have traces of the past and no traces of the future), the asymmetry of causation (we have an impact on future events with no possibility of having an impact on the past), and the asymmetry between the fixed past and the open future, To this end, the approaches of Boltzmann, Reichenbach (and his followers), and Albert are analysed. It is argued that we should look for alternative approaches instead of this, namely we should consider a temporally asymmetrical physical theory or seek a source of the asymmetry of time in metaphysics. This second approach may even turn out to be complementary if only we accept that metaphysics can complement scientific research programmes.

## 1. Introduction: The Asymmetry *of* Time and the Asymmetry *in* Time

One of the most fundamental features of our world is that it is strongly temporally asymmetrical: as our experience shows, we have traces of the past in our memory and around us and no traces of the future; we can have an impact on future events with no possibility of having such an impact on the past; meanwhile, the future seems to be open, while the past is fixed and cannot be changed. The problem of the direction or the asymmetry *of* time itself consists in answering the question of whether the world is truly temporally asymmetrical and in finding an explanation of what is responsible for this asymmetry (Some philosophers, such as Mehlberg [1], Horwich [2], and Huw Price claim that the world is temporally symmetrical due to the temporal symmetry of fundamental physical interactions; according to them, an alleged temporal asymmetry is a product of our awareness. Adherents of such a view should explain where such an illusion comes from: a far from easy task which I will show by means of the example of Price in the third section of this paper).

Two clarification are needed here: firstly, after Sklar [3,4,5,6], I distinguish between the asymmetry *of* time and asymmetry *in* time. That the causation is future directed and we find only traces of the past but can affect only the future, and that the past cannot be changed while the future can be, seem to be essential features *of* time itself, and not this or any other particular process occurring *in* time. Like many others, I eat my dessert after my main courses and go to bed after sunset, but these temporally asymmetrical processes do not constitute the asymmetry *of* time itself; they are only some processes which are asymmetrical *in* time and tell us nothing about time itself.

In contrast to such processes which are asymmetrical *in* time, the three features mentioned in the beginning seem to constitute essential features *of* time itself and require explanation. This is a second point which I feel it is important to emphasise. The asymmetry *of* time constituted by these three asymmetries is *fundamental* for us and every explanation of the directionality *of* time since Boltzmann’s *Lectures on Gas Theory* [7] (Boltzmann [7]. See also Sklar [3,4,6]; and Gołosz [8,9,10]) has stemmed from our attempts to understand these asymmetries, and that is why every plausible theory of the direction of time should explain them in a credible way.

What do I mean by plausible explanation? We have perfect examples of such explanations in physics: we are able to explain that light is an electromagnetic wave and what constitutes the difference between “up” and “down” are gravitational forces (or the structure of spacetime in the general theory of relativity (GTR)). That we can try to explain the direction of time in a similar way to the fact that the difference between “up” and “down” is explained by gravitational forces was suggested by Boltzmann himself [7], as I will recall in the next section.

We believe in physics and that it should explain all of the essential characteristics of the world and the three fundamental asymmetrical features of the world as well. Unfortunately, it transpires that fundamental interactions, that is, strong, electromagnetic and gravitational interactions, are time reversal invariant and as such they cannot explain these asymmetries. Although weak interactions are not time reversal invariant, they are unable to explain this asymmetry as well because of two reasons:

(1) they do not take part in everyday processes which are temporally asymmetrical, when we are, for example, writing, reading, or doing some task [3,6,11];
(2) weak interactions are only *feebly* temporally asymmetrical, that is, for any weak process, a time-reversed sequence of time-reversed states is possible, although it can have a different probability, while time is *strongly* temporally asymmetrical, that is we have *no* cases of backward causations, *no* traces of the past and *no* possibility of influencing the past [8,9,10,12].

That is why weak interactions are unable to ground and explain the asymmetry of time and the processes involving weak interactions should be qualified as only processes which are asymmetrical *in* time.

Although the laws of physics do not provide us with enough temporal asymmetry, one could insist that we have enough asymmetry in the world as it is described by physics to explain the asymmetry *of* time after all: if not in physical laws than perhaps we should look for the direction of time in temporary asymmetrical processes *de facto*. Of course, the process of the increase of entropy has been the most plausible candidate for any explanation of the direction of time since the second half of the 19th century (Whenever the term entropy is employed in this paper, it should be read in the sense of Boltzmann’s understanding of entropy). Contrary to this, using different examples in the second section of this paper, I would like to show that despite these high hopes, the process of the increase of entropy is unfortunately not up to the task, that is, it explains none of the three abovementioned temporal asymmetries. This will be demonstrated by means of the well-known examples from Boltzmann, Reichenbach (and his followers), and Albert, namely that the increase of entropy is unable to explain the asymmetry of traces, the asymmetry of causation, and the asymmetry between the fixed past and the open future, and it should only be treated as a process that is asymmetrical *in* time.

## 2. The Increase of Entropy as a Process Asymmetrical *in* Time

As is well-known, Boltzmann hoped that the process of the increase of entropy would explain the manner in which we experience the direction of time in precisely the same manner that gravity explains the difference between the directions of up and down:

“*One can think of the world as a mechanical system of an enormously large number of constituents, and of an immensely long period of time, so that the dimensions of that part containing our own “fixed stars” are minute compared to the extension of the universe; and times that we call eons are likewise minute compared to such a period. Then in the universe, which is in thermal equilibrium throughout and therefore dead, there will occur here and there relatively small regions of the same size as our galaxy (we call them single worlds) which, during the relative short time of eons, fluctuate noticeably from thermal equilibrium, and indeed the state probability in such cases will be equally likely to increase or decrease. For the universe, the two directions of time are indistinguishable, just as in space there is no up or down. However, just as at a particular place on the earth’s surface we call “down” the direction toward the centre of the earth, so will a living being in a particular time interval of such a single world distinguish the direction of time toward the less probable state from the opposite direction (the former toward the past, the latter toward the future). By virtue of this terminology, such small, isolated regions of the universe will always find themselves “initially” in an improbable state. This method seems to me to be the only way in which one can understand the second law—the heat death of each single world—without a unidirectional change of the entire universe from a definite initial state to a final state*.”

[7] (pp. 402–403)

What reduction does Boltzmann have in mind? *Reduction by definition* or *scientific reduction*? One might speculate that he did not think about reduction *by definition* because in such a case—as noticed by Eddington [13] (p. 93)—the second law of thermodynamics became an analytic truth which is always truly independent of the real course of processes in the world. I do not suppose that Boltzmann wanted to transform the second law of thermodynamics into a tautology.

Yet if he had in mind *scientific reduction*, then—as noticed by Sklar [3] (chapter V)—he should explain why we have traces of the past and no traces of the future; why we can have an impact on future events with no possibility of having an impact on the past; and why the future seems to be open, while the past is fixed and cannot be changed in a similar way as we explain the difference between “up” and “down” with the aid of gravitational forces. Unfortunately, he did not do so and I will show later that the attempts of Reichenbach and his followers to explain the asymmetry of our knowledge concerning the past and future, that is, the first asymmetry, failed as well.

What is more, when we assess Boltzmann’s attempt to explain the asymmetry *of* time, we should take into account the real status of the second law of thermodynamics and the temporal symmetry of statistical mechanics (SM). This latter says—as noticed by Boltzmann—that the entropy of a physical system which is in a state of thermal equilibrium can spontaneously *fluctuate* to more ordered states and that physical systems that are not initially in a state of thermal equilibrium will *evolve* with a great probability to more probable states, that is, to states with greater entropy. Such a theory takes for granted the *dynamical* evolution of physical systems and the flow of time: in the course of the flowing of time, the systems can fluctuate, firstly decreasing and then increasing its entropy. Yet this means that we assume the existence of the flow of time and, in consequence, the existence of an objective arrow of time connected with the passage of time which is not explained by Boltzmann’s reduction and makes his reduction redundant. In turn, if we do not assume a dynamical evolution of physical systems based on the flow of time, Boltzmann’s magnificent explanation of the statistical behaviour of these systems with the aid of occurring spontaneous fluctuations becomes incomprehensible.

I have critically analysed Boltzmann’s approach to the arrow of time. For philosophers, the attempts of Reichenbach [14], and his followers (particularly Smart [15] and Grünbaum [16]) are perhaps better known. According to the approach initiated by Reichenbach, if we find some traces of the past, such as footprint shaped marks on the beach, we can infer from this that “at some earlier time an interaction took place, that a person‘s steps caused the ordered state of the sand” [14] (p. 151) because “this orderliness is bought at the expense of an increased disorderliness (metabolic depletion) of the pedestrian who made it” [15] (p. 469) or—using Grünbaum’s words—“we can reliably infer a past interaction of the system with an outside agency from a present ordered or low entropy state” [16] (p. 235).

However, as highlighted by Earman [17], we do not have to appeal to entropy considerations to infer our knowledge about the past from traces: traces give us much more information which is more precise than that which would follow from entropy considerations—these, at best, would only say about the past interaction of the system whose entropy is lower than it should be with some external system so as to produce a greater order of the sand (See [17] (pp. 43–45)). Secondly, it can be seen from the above citations (from Reichenbach, Grünbaum, and Smart) that an assumption about some *asymmetric causal interaction* is involved in every such inference, which means that this inference is in fact based on temporal asymmetric causation (See [17] (pp. 41–42)), and since causal theories of time direction have failed (as noticed below), this reasoning also fails. Thirdly, there are well known cases—for example, when a bomb is dropped on a city—when the formation of subsystems of temporarily higher entropy than their surroundings form traces which are easily readable for us (See [17] (p. 40)). All this shows that the entropic approach to the asymmetry of our knowledge adopted by Reichenbach, Smart, and Grünbaum is unsuccessful; we have to appeal to the asymmetry of causation and apply a causal theory of time to explain the asymmetry of our memory but this approach does not work.

This is perhaps not the best place for a critique of causal theories of time direction, I would like only to note that it is reasonable to assume that physical interactions are involved in all causal relations and because strong, electromagnetic and gravitational interactions are time reversal invariant, the causal theories of time are unable to distinguish between the past and the future [3,8,9].

As a response to such objections, one might try to apply the strategy of biting the bullet and assume that causal relations are reduced to thermodynamic processes as well. A prominent adherent of such an approach is David Albert (See Albert [18]. See also Loewer [19] and Frisch [20]. Albert did not analyze Earman’s critique of entropic approach in his book). Albert claimed that causal asymmetry is grounded in the same processes that give rise to the second law of thermodynamics, first of all in a low-entropy constraint on the initial state of the universe, which is known as the *Past Hypothesis* (PH) According to the PH, “the world first came into being in whatever particular low-entropy highly condensed big-bang sort of macrocondition it is that the normal inferential procedures of cosmology will eventually present to us”. [18] (p. 96).

Generally speaking, David Albert claims that according to SM and PH, possible macroevolutions are much more restricted toward the past than toward the future and this is responsible for the temporal directedness of our own capacity to *acquire information* about the world and to *influence causally* the occurrence of future events but not past events [18] (p. X, chapter 6). To understand the problem of how PH works, we should—according to Albert—focus our attention on macro events such as billiard ball collisions in a given collection which behave—Albert claims—in a temporally asymmetrical way:

“*Think (to begin with) of the collection of billiard balls we were talking about before. Suppose that some particular one of those balls (ball number 5, say) is currently stationary. Then suppose (and this is what is going to stand out—in the context of this extremely simple example—for a past hypothesis) that that same ball is somehow known to have been moving ten seconds ago*.”

[18] (p. 126)

It follows from the laws of mechanics that ball 5, which is currently stationary but *on Albert’s assumption* was moving ten seconds ago, had to have been involved in a collision in the past 10 s. On these assumptions, he claims ([18] (pp. 126–128)), that whereas, on the one hand, there are obviously any number of hypothetical alterations of the present condition of the balls in the set which would alter the facts about whether ball number 5 is to be involved in a collision over the next ten seconds or not, there can be, on the other hand, *no* hypothetical alterations in the present condition of this set of balls, unless they involve hypothetical alterations in the present velocity of ball number 5 *itself,* which would alter the facts about whether or not ball number 5 *had been* involved in a collision over the *past* ten seconds. That is why “there are (as it were) a far wider variety of potentially available *routes to influence* over the future of the ball in question here, there are a far wider variety of what we might call *causal handles* on the future of the ball in question here, under these circumstances, than there are on its past” [18] (p. 128).

Here—and which is important—“under these circumstances” means that it is taken for granted that ball 5 is somehow known to *have been moving ten seconds ago* [18] (pp. 126–128). In this example, the condition that ball 5 was moving ten seconds ago plays the role of a PH while—as Albert emphasizes—we do not have its counterpart concerning the future.

Now, the important question arises as to whether Albert managed to explain the asymmetry *of* time, that is, the asymmetry of how we *acquire information* about the world and the asymmetry of how we *influence causally* the occurrence of future events but not of past events. Unfortunately, the answer must be in the negative: assuming that ball 5 in his example is somehow known to *have been moving ten seconds ago* while not assuming a symmetrical postulation concerning the future, Albert *introduced the temporal asymmetry* into the process analysed in his argument instead of explaining it. More specifically, he assumed the fundamental asymmetry between the fixed past and the open future, that is, this very asymmetry which he should explain and in this way his reasoning begs the question. This mistake is even more perplexing if one takes into account the fact that Albert begins his book with the strong declaration that “[t]his book is intended both as an elementary introduction and as an original contribution to the development of a scientific account of the distinction between the past and the future” (Albert [18] (p. viii). Hemmo and Shenker [21] also claim, as does the author of this paper [8,9,10], that the second law of thermodynamics and the past hypothesis in statistical mechanics cannot yield arrows of time since the second law of thermodynamics and PH already assume an arrow of time).

Another fundamental objection can be raised against Albert’s approach and all those that try to reduce the asymmetry of our knowledge and asymmetry of causation to the second law of thermodynamics. Namely, according to SM, it is possible in every physical system during some period of time that entropy will be constant or even decrease as an effect of thermodynamic fluctuations. Should we then believe that causal relations in the first case will be temporally symmetric and—in the second one—change its temporal direction? Should we believe that our knowledge concerning the past and the future in the first case becomes temporally symmetric?

In turn, Frisch [20] (Section 3) highlights the obvious fact that thermodynamic asymmetry often results in the destruction of records or traces of the past and there are many “human-scale” macroevents that leave no or only very few traces in their futures. If, for example, the wind wipes out the footprint-like traces in the sand and traces of ancient civilizations have vanished under layers of sand and soil, it can be argued that one central role played by the thermodynamic arrow is as a destroyer of macrorecords and macrotraces rather than as their creator. In consequence, we cannot treat the increase of entropy as being responsible for the fact that we have traces of the past and no traces of the future.

Frisch [20] (Section 3) also criticizes Albert’s claim that, according to SM and PH, possible macroevolutions are much more restricted toward the past than toward the future, that is—in Loewer’s [19] terminology—the claim that a possible macroevolution has a “tree structure”. Frisch indicates that, while there really is a macrobranching of many physical systems toward the future, thermodynamic considerations imply that there is also a possible widespread reconvergence of possible macrohistories. For example, at the cosmological level, even though the initial state of the universe might not determine the large-scale distribution of matter, different cosmological macrohistories will converge toward the final equilibrium state. Similarly, Frisch shows that there is a convergence at the level of “human-sized” macrosystems: for example, in the paradigmatic case of a container half filled with gas, *no matter which part of the container* the body of gas initially occupies, the gas will spread after the partition is removed until it is uniformly distributed throughout the whole container. All this suggests that possible macroevolutions may exhibit an upside-down tree structure.

## 3. Final Remarks

I have tried to show that the thermodynamic explanation of the asymmetry of time is implausible because the process of the increase of entropy does not explain why we have traces of the past in our memory and around us and no traces of the future; why we can have an impact on future events with no possibility of having an impact on the past; and why the future seems to be open, while the past is fixed and cannot be changed. Thus, if I am right in adopting this line of argumentation, one can conclude that the increase of entropy as it is described by the second law of thermodynamics is only a process which is asymmetrical *in* time and in no way helps us to explain the asymmetry *of* time itself.

Perhaps, in such a situation where the strong, electromagnetic and gravitational interactions are time reversal invariant and the increase of entropy does not explain the asymmetry *of* time, we should maintain that time has no direction and following Price [22], for example, assume that this essential component of temporal asymmetry, namely causal asymmetry, reflects an asymmetry in us rather than an asymmetry in the external world. He suggested that causal asymmetry may be conventional, or perspectival, that is, not an objective aspect of the world, but a kind of projection of our own internal temporal asymmetry as agents who act in the world with the thermodynamic gradient:

“*From an objective standpoint, very crudely, an agent is simply a natural system which correlates inputs with outputs. The inputs are environmental data and the outputs are behavior. The details of these correlations vary with the agent’s internal state, and this too may vary in response to inputs. The terms “input” and “output” assume a temporal direction, of course, but this is inessential. From an atemporal viewpoint what matters is that events on one temporal side of the box get correlated with events on the other side. It does not matter that one side is thought of as earlier and the other later. From a sufficiently detached perspective, then, deliberation appears broadly symmetric in time—an agent is simply a “black box” which mediates some otherwise unlikely correlations of this kind. Certainly, the operations of working models may depend on temporal asymmetry, in the way that actual agents require the thermodynamic gradient, but it is possible to characterize what such a system does, at least in these very crude black box terms, without specifying a temporal direction*.”

[22] (p. 168)

Is it really a plausible solution? I do not believe so. Let us consider Price as an agent who is writing his book [22]—this is our “black box”—from an atemporal point of view, which is preferred by him (see [8] (p. 180)). Then, on one temporal side of the box, we receive Price, who is collecting more and more material for his book and analysing it. On the other side, however, we receive the book and philosophers who are reading it. Yet why do we have traces of Price’s work and rising traces of the reading of it (for example, in the form of a growing number of citations) on only one temporal side? The thermodynamic gradient, as I have shown in the former section, does not explain the asymmetry of traces and Price, unfortunately, does not add anything new to this subject. We are also left in the dark as to why Price is only able to answer objections raised to his arguments on one temporal side of “black box”, that is precisely the one which is *later*. Therefore, unfortunately, Price’s solution appears to be highly implausible.

Thus, it seems that the idea that time has no direction is a far from tempting one. Now, however, an important question arises: if time really is asymmetric, how can we explain such a fundamental property of our world which is the asymmetry *of* time? I suggested in my former paper [10] that there are two possible ways we can look for solutions to this conundrum: the first is that the future quantum gravity which we are looking for will be a time-asymmetric theory and perhaps such a theory would be able to explain the asymmetry *of* time. The second possibility is that we should seek the origin of the asymmetry *of* time in metaphysics rather than in physics.

Thus, Penrose [23] (pp. 345, 350–353) suggests that our sought-for quantum gravity must be a time-asymmetric theory. To the possible objection that his postulated theory should correspond to GTR in the “classical” level and GTR is itself time-symmetric, Penrose replies that although the separation between dynamical equations and initial (or boundary) conditions has historically been of vital importance, his sought-for theory should dissolve away this separation. That is, he suggests that future quantum gravity should absorb PH into its main body in some way, and should not treat it as something external. Such a move would, of course, be at odds with our present methodological rules as they have been assumed since Newton but—as was noticed by Laudan [24]—the methodological rules can change when science develops and new theories are created, so nobody can a priori exclude Penrose’s proposal as implausible.

The second—metaphysical—approach to the asymmetry of time was developed by the author of this paper [8,12,25,26,27]. According to this approach, temporal asymmetry is introduced into our world by the way it exists, that is, by *dynamic existence*, which is a generalisation of the notion of *becoming*. That we should include becoming into our image of the world has been advocated by, inter alia, Ellis [28], Smolin [29], and Rovelli [30]. Rovelli, for example, wrote:

“*Physics (if not science in general) is a theory about how things happen. Its core, since ancient astronomy, Galileo, Kepler and Newton, all the way to quantum field theory and general relativity, is the description of motion, evolution, change, becoming*.”

[30] (p. 1331)

In turn, Smolin adds a number of interesting remarks:

“*If space is emergent, does that mean that time is also emergent? If we go deep enough into the fundamentals of nature, does time disappear? In the last century, we have progressed to the point where many of my colleagues consider time to be emergent from a more fundamental description of nature in which time does not appear*.

*I believe—as strongly as one can believe anything in science—that they are wrong. Time will turn out to be the only aspect of our everyday experience that is fundamental. The fact that it is always some moment in our perception, and that we experience that moment as one of a flow of moments, is not an illusion. It is the best clue we have to fundamental reality*.”

[29] (p. xxxi)

“*We have to find a way to unfreeze time—to represent time without turning it into space. I have no idea how to do this. I cannot conceive of a mathematics that doesn’t represent a world as if it were frozen in eternity*.”

[29] (p. 257)

If mathematics tends to represent a world as if it were frozen in eternity, it is a strong indication, I think, that we should look for our solution to unfreezing time elsewhere. My choice is metaphysics: the main thesis of my position, which is called *dynamic presentism*, says that all of the objects that our world consists of, exist dynamically. The notion of dynamic existence is treated in my approach as a primitive one which is roughly characterised by the following set of three postulates:

(i) the notion of dynamic existence is tensed;
(ii) things that dynamically exist endure;
(iii) events (which are acts of acquiring, losing, or changing properties by dynamically existing things and their collections) dynamically exist in the sense of coming to pass (See [12] (pp. 41–42) and [27] (Section 3). Things endure when they persist over time, keeping their strict identity).

The first postulate means that we can say about what *dynamically existed* (the past), what *dynamically exist* (the present), and what *will dynamically exist* (the future), that is, we are able to differentiate between the past, the present, and the future.

Such a metaphysical theory introduces dynamics into the world and explains all of the fundamental temporal asymmetries: firstly, the future is (or seems to be) open, while the past is fixed because events, which are acts of acquiring, losing, or changing properties by dynamically existing things and their collections, dynamically exist in the sense of coming to pass. Secondly, the enduring things—one can say metaphorically—dynamically exist toward the future carrying traces of the past interactions and thanks to this, in spite of the symmetry under time reversal of physical interactions (modulo weak interactions, of course), convey traces of the past into the future. Thirdly, and because of the same reason, things can impact on future events with no possibility of having an impact on the past.

One can perhaps treat the fact that it is metaphysical and not physical as a drawback of this last explanation of asymmetry *of* time, and that it introduces a piece of hard metaphysics into the heart of our knowledge about the physical world. I think, however, that such an objection would be too strong. Firstly, we know after the demise of logical positivism that metaphysical ideas can be introduced into scientific research programmes [31,32,33]. Secondly, it transpires that the situation is not so bad and that not only do the two proposed approaches—the physical and the metaphysical—not contradict one another, but even then, it can turn out that they are deeply complementary. Namely, in some approaches to quantum gravity, for example, in causal dynamical triangulation [34,35,36], temporal asymmetry and dynamics are strongly desired because their authors assume that spacetime has a built-in arrow of time which is needed to distinguish between causes and effects and in turn causal time-asymmetric processes are necessary to treat the spacetime as emerging dynamically as a four-dimensional object. Unfortunately, the authors of this approach do not explain the origin of dynamics and the asymmetry of causation, and thus the kind of metaphysics introduced above, if added, can be treated as a source of both these lacking components [10,12].

I think that it is by no means accidental that both the proposals for solving the problem of the asymmetry of time introduced above introduce new methodological approaches: the fact that we have so many doubts concerning such a fundamental property of our world means that we do not understand it and perhaps we should change the way we think about it. After all, former revolutions in physics—such as those connected with Newton, Einstein, or quantum mechanics—involved essential changes in our thinking about science itself [23,33], so we can expect that some changes will be necessary in the future as well.

## References

[1] Mehlberg H., Cohen R.S. (1980). Time, Causality and the Quantum Theory.

[2] Horwich P. (1987). The Asymmetries in Time: Problems in the Philosophy of Science.

[3] Sklar L. (1974). Space, Time, and Spacetime.

[4] Sklar L. (1993). Physics and Chance: Philosophical Issues in the Foundations of Statistical Mechanics.

[5] Sklar L., Savitt S. (1995). Time in Experience and in Theoretical Description of the World. Time’s Arrow Today.

[6] Sklar L., Borchert D.M. (2005). Physics and the Direction of Time. Encyclopedia of Philosophy.

[7] Boltzmann L., Brush S. (1964). Lectures on Gas Theory.

[8] Gołosz J. (2011). Upływ Czasu i Ontologia.

[9] Gołosz J. (2017). Weak Interactions: Asymmetry of Time or Asymmetry in Time?. J. Gen. Philos. Sci..

[10] Gołosz J. (2017). The Asymmetry of Time: A Philosopher’s Reflections. Acta Phys. Pol. B.

[11] Feynman R. (1967). The Character of Physical Law.

[12] Gołosz J., Hanna P. (2020). In Defence of a Dynamic View of Reality. An Anthology of Philosophical Studies.

[13] Eddington A.S. (1929). The Nature of Physical World.

[14] Reichenbach H., Reichenbach M. (1956). The Direction of Time.

[15] Smart J.J.C., Borchert D.M. (2005). Time. Encyclopedia of Philosophy.

[16] Grünbaum A. (1973). Philosophical Problems of Space and Time.

[17] Earman J. (1974). An Attempt to Add a Little Direction to ‘The Problem of the Direction of Time’. Philos. Sci..

[18] Albert D.Z. (2000). Time and Chance.

[19] Loewer B., Price H., Corry R. (2007). Counterfactuals and the Second Law. Causality, Physics, and the Constitution of Reality: Russell’s Republic Revisited.

[20] Frisch M., Hüttemann A., Ernst G. (2010). Does a low-entropy constraint prevent us from influencing the past?. Time, Chance, and Reduction: Philosophical Aspects of Statistical Mechanics.

[21] Hemmo M., Shenker O., Dolev Y., Roubach M. (2016). The Arrow of Time. Cosmological and Psychological Time.

[22] Price H. (1997). Time’s Arrow & Archimedes’ Point. New Directions for the Physics of Time.

[23] Penrose R. (1989). The Emperor’s New Mind.

[24] Laudan L. (1984). Science and Values.

[25] Gołosz J. (2015). How to Avoid the Problem with the Question about the Rate of the Time’s Passage. Rev. Port. Filos..

[26] Gołosz J. (2018). Presentism and the Notion of Existence. Axiomathes.

[27] Gołosz J. (2020). Brute Past Presentism, Dynamic Presentism, and the Objection from Being-Supervenience. Axiomathes.

[28] Ellis G.F.R. (2006). Physics in the Real Universe: Time and Spacetime. Gen. Relativ. Gravit..

[29] Smolin L. (2013). Time Reborn: From the Crisis in Physics to the Future of the Universe.

[30] Rovelli C. (2019). Neither Presentism nor Eternalism. Found. Phys..

[31] Lakatos I., Lakatos I., Musgrave A. (1970). Falsification and the Methodology of Scientific Research Programmes. Criticism and the Growth of Knowledge.

[32] Laudan L. (1977). Progress and its Problems: Towards a Theory of Scientific Growth.

[33] Gołosz J. (2011). Science, Metaphysics, and Scientific Realism. Pol. J. Philos..

[34] Ambjørn J., Görlich A., Jurkiewicz J., Loll R. (2008). Planckian Birth of the Quantum de Sitter Universe. Phys. Rev. Lett..

[35] Ambjørn J., Jurkiewicz J., Loll R. (2008). The Self-Organizing Quantum Universe. Sci. Am..

[36] Ambjørn J., Görlich A., Jurkiewicz J., Loll R., Ashtekar A., Petkov V. (2014). Quantum Gravity via Causal Dynamical Triangulation. Springer Handbook of Spacetime.

